# Short-interval intracortical inhibition is not affected by varying visual feedback in an isometric task in biceps brachii muscle

**DOI:** 10.3389/fnhum.2013.00068

**Published:** 2013-03-08

**Authors:** Timo Rantalainen, Ashleigh Weier, Michael Leung, Chris Brandner, Michael Spittle, Dawson Kidgell

**Affiliations:** ^1^Centre for Physical Activity and Nutrition Research, School of Exercise and Nutrition Sciences, Deakin UniversityMelbourne, VIC, Australia; ^2^Department of Health Sciences, University of JyväskyläJyväskylä, Finland; ^3^Centre for Exercise and Sports Science, School of Exercise and Nutrition Sciences, Deakin UniversityMelbourne, VIC, Australia

**Keywords:** transcranial magnetic stimulation, primary motor cortex, task specificity, force gradation, motor control

## Abstract

Short-interval intracortical inhibition (SICI) of the primary motor cortex (M1) appears to play a significant role in skill acquisition. Consequently, it is of interest to find out which factors cause modulation of SICI.

**Purpose:** To establish if visual feedback and force requirements influence SICI.

**Methods:** SICI was assessed from 10 healthy adults (5 males and 5 females aged between 21 and 35 years) in three submaximal isometric elbow flexion torque levels [5, 20, and 40% of maximal voluntary contraction (MVC)] and with two tasks differing in terms of visual feedback. Single-pulse and paired-pulse motor-evoked potentials (MEPs), supramaximal M-wave, and background surface electromyogram (sEMG) were recorded from the biceps brachii muscle.

**Results:** Repeated measures MANOVA was used for statistical analyses. Background sEMG did not differ between tasks (*F* = 0.4, *P* = 0.68) nor was task × torque level interaction observed (*F* = 1.2, *P* = 0.32), whereas background sEMG increased with increasing torque levels (*P* = 0.001). SICI did not differ between tasks (*F* = 0.9, *P* = 0.43) and no task × torque level interaction was observed (*F* = 2.3, *P* = 0.08). However, less SICI was observed at 40% MVC compared to the 5 and 20% MVC torque levels (*P* = 0.01–0.001).

**Conclusion:** SICI was not altered by performing the same task with differing visual feedback. However, SICI decreased with increasing submaximal torque providing further evidence that SICI is one mechanism of modulating cortical excitability and plays a role in force gradation.

## Introduction

It is well-established that the primary motor cortex (M1) is able to modify its function in response to chronic activity. This response has been termed “plasticity” and involves reorganization of neural assemblies that control movement (Pascual-Leone et al., 1995; Ljubisavljevic, 2006; Butler and Wolf, 2007). The plastic change of the cortex may be assessed non-invasively, e.g., by using transcranial magnetic stimulation (TMS) and is manifested in changes in the area of the cortical representation and in changes in cortical excitability (Pascual-Leone et al., 1995; Ljubisavljevic, 2006). Changes in GABAergic (GABA_A_) cortical inhibitory interneuron activity can be assessed by measuring short-interval intracortical inhibition (SICI). Decreases in SICI have been shown to be associated with a single bout of skill training (Perez et al., 2004; Cirillo et al., 2011; van den Berg et al., 2011) and in association with short-term (4 weeks) strength training interventions (Goodwill et al., 2012; Latella et al., 2012; Weier and Kidgell, 2012; Weier et al., 2012). Consequently, SICI has been suggested to play a particularly important role in the modulation of neural function during skill acquisition (Ljubisavljevic, 2006).

Although SICI seems to play an important role in task acquisition, little is known regarding tasks that affect SICI acutely. Modifying force demands of an isometric task have indicated that SICI is decreased with increased voluntary activation, albeit with no further reductions in SICI when force levels were above 25% of maximal voluntary contraction (MVC) (Zoghi and Nordstrom, 2007; Ortu et al., 2008). Also, coactivation of synergistic muscles has been reported to decrease SICI (Devanne et al., 2002). Other possible tasks which could have affected SICI acutely, but have been shown not to have an effect, include different types of grips (Davare et al., 2008; Kouchtir-Devanne et al., 2012) and activation of homologous muscle (Christova et al., 2006; Howatson et al., 2011).

Studies on the effects of visuomotor feedback on task performance have shown that force fluctuations around a target force level increase when visual feedback is provided compared to when no visual feedback is provided (Tracy, 2007; Tracy et al., 2007). Moreover, modifying the visual feedback scale has also been shown to affect force variation with an increase in force fluctuations with increased precision of visual information scale in an isometric force maintenance task (of note an initial decrease in fluctuations was reported with increasing precision of visual information scale, when the visual information scale gradation was relatively rough) (Sosnoff and Newell, 2006). In agreement, modifying visual feedback in an isometric task has been found to shorten the silent period caused by TMS (Hess et al., 1999; Pearce and Kidgell, 2009, 2010). Moreover, a single bout of visuomotor training has been shown to lead to decreased SICI (Cirillo et al., 2011). However, it is not known whether SICI is modified acutely by visual feedback.

It is unclear what kind of interplay visual feedback and force requirements have on SICI. Therefore, the purpose of the present study was to investigate SICI with acute variations in force requirements and with different levels of visual feedback. Based on existing literature, it was hypothesized that SICI would decrease with increasing voluntary force output (Zoghi and Nordstrom, 2007; Ortu et al., 2008) and that changing visual feedback would change SICI (Hess et al., 1999; Pearce and Kidgell, 2009, 2010).

## Materials and methods

### Participants

Ten healthy, right-hand dominant individuals with no history of neurological disease volunteered to participate in the study (5 men and 5 women, aged between 21 and 35 years). All participants provided written, informed consent to the procedures of the study, which conformed to the Declaration of Helsinki and were approved by a Human Research Ethics Committee.

### Experimental protocol

Participants were required to attend the laboratory on two consecutive days. On the first day, maximal isometric elbow flexion torque (MVC) was obtained. All other measurements were carried out on the second day. Prior to initiating the measurements on either day, the participants were asked to warm up with three sets of eight repetitions of a biceps curl with a 4 kg dumbbell for the right limb only.

The second measurement day comprised determining active motor threshold (AMT) of TMS and measuring MEPs from the right arm biceps brachii muscle in two tasks at three different submaximal torque levels. In total, participants were required to complete 20 isometric contractions in a randomized order at each target torque level for both types of visual feedback, amounting to a total of 120 trials. The 20 contractions at each isometric force level comprised 15 paired-pulse TMS trials and five single-pulse TMS trials. In addition, nine resting supramaximal M-waves were recorded from the biceps brachii and brachioradialis muscles of the right arm. Two of the M-waves were recorded at the beginning of the experiment, two at the end, and the other five were randomized within the TMS trial blocks. A purpose made Excel macro was used to randomize the TMS trials in blocks of five contractions.

### Maximal voluntary contraction

MVCs were obtained to represent the maximal voluntary effort. Participants were seated in an isokinetic dynamometer (Biodex System 4 Pro, Biodex Medical Systems, Shirley, USA) with their right elbow positioned at 90° of elbow flexion, forearm in a horizontal orientation, and with the hand supinated. The torque was sampled at 1000 Hz. Participants were required to pull against the dynamometer handle and produce a gradual increase in flexion torque to its maximum. Once the maximum torque was obtained it was held for a subsequent 3 s. The participants were given verbal encouragement, and visual feedback of the torque exerted was provided via the Biodex monitor which was located at eye level ~1.5 m away from the participant. The maximum of the three trials was recorded as the participant's MVC torque. This value was used to determine the target torque levels (5, 20, and 40% MVC torque) to be maintained during TMS trials.

### Torque gradation

Isometric contractions were performed for three target torque levels (5, 20, and 40% of MVC torque) and for each of the two tasks (low and high visuomotor tasks).

### Visuomotor task

The participant was required to produce a constant isometric torque level with the aid of on-line torque feedback, as determined by the specific task requirements. The participants were asked to reach the target torque level in their preferred manner and once the participant had maintained a relatively constant target torque level for a minimum of 1 s, the TMS pulse was applied, by manually triggering the stimulator. To produce two tasks with differing visual feedback, the scaling of the on-line torque trace was modified. Two scales, low from 0 to 150% of target torque and high from 90 to 110% were used, which resulted in a 7.5 fold scaling difference between the tasks. The height and width of the visual field used to provide the torque feedback was held constant. The feedback was modified by either (1) plotting the torque scale from 0 to 150% of the torque target with a horizontal line at the target torque level; or (2) plotting the torque scale from 0.9 times of the target torque level to 1.1 times the target torque level and highlighting the area between 0.95 times the target and 1.05 times the target. In this context, the first form of visual feedback was classified as low visuomotor feedback, whilst the second condition was classified as high visuomotor feedback.

### Electromyography

Bipolar surface electromyogram (sEMG) were collected from the biceps brachii and brachioradialis muscles using Ag-AgCl electrodes. All sEMG signals (MEPs) were sampled at 1000 Hz and collected on a PC running commercially available software PowerLab 8 (ADinstruments, Australia) via a laboratory analog-digital interface (PowerLab 8/30, ADinstrument, Australia) for later off-line analysis. sEMG signals were filtered and amplified (1000 ×) with bandpass filtering between 10 and 500 Hz.

### Transcranial magnetic stimulation

TMS was applied over the cortical representation of the right biceps brachii muscle group, using a figure-eight coil (70 mm diameter) attached via a BiStim unit to two Magstim 200^2^ stimulators (Magstim Co, UK). The center of the TMS coil was positioned over the “hot spot” and held tangential to the skull in an anterior-posterior orientation, inducing a posterior-anterior current on the cortex for activating the right biceps brachii muscle. Sites near the estimated motor area of the biceps brachii were explored and marked to determine the site in which the largest MEP could be evoked during a low level contraction (10% MVC). The AMT was determined as the minimum stimulus intensity required to elicit a MEP in the right biceps brachii of at least 200 μV in three out of five consecutive trials during low level voluntary elbow flexion (10% MVC). AMT was expressed relative to 100% maximum stimulator output (MSO), and the stimulus intensity was altered in 1% increments of MSO throughout this process until the appropriate threshold level was achieved (Kidgell and Pearce, 2010).

### Short-interval intracortical inhibition

SICI was assessed using a paired-pulse protocol that consisted of a subthreshold conditioning stimulus that preceded a suprathreshold test stimulus by 3 ms. The test stimulus was set at 120% of AMT, whereas the conditioning stimulus was set at 80% of AMT (Kujirai et al., 1993; Lackmy and Marchand-Pauvert, 2010). Five single-pulse stimuli and 15 paired-pulse stimuli were applied during each of the tasks and at each of the torque levels in a randomized order at a minimum of 12 s apart.

### M-waves

Direct muscle responses were obtained from the right biceps brachii muscle by supramaximal electrical stimulation (pulse width 200 μs) of the brachial plexus (Erbs point) under resting conditions (DS7A, Digitimer, UK). The site of stimulation that produced the largest M-wave was located by positioning the bipolar electrodes in the supraclavicular fossa. An increase in current strength was applied to the brachial plexus from below the participant's threshold until there was no further increase observed in the amplitude of the sEMG response (M_MAX_). To ensure maximal responses, the current was increased an additional 20%. Two M-waves were recorded at the beginning and at the end of the protocol in addition to the five stimuli randomized into the TMS protocol. The average of the nine stimuli were then used to establish and report M_MAX_.

### Data analysis

All analysis was conducted off-line with custom written Octave (Octave 3.2.4, http://www.octave.org) scripts.

Peak to peak amplitude and 30 ms root mean square (RMS) amplitude were automatically analysed from the M-wave separately for biceps brachii and brachioradialis. The analysis was conducted by searching for the lowest and highest peaks in the EMG trace from a 30 ms epoch, which started 10 ms after the triggering of the percutaneous nerve stimulation. The lowest value was subtracted from the highest value to produce peak to peak M-wave value. Thereafter, the center of the 30 ms RMS epoch was set to the mean between the highest and the lowest peak of the M-wave and RMS amplitude calculated from this epoch. In case that the beginning of the RMS epoch would have been less than 5 ms from the trigger, the beginning of the epoch was set at 5 ms from the trigger.

Background sEMG of 500 ms RMS amplitude was automatically analysed from the biceps brachii and brachioradialis sEMGs from an epoch that began 500 ms prior to the triggering of the TMS. The calculated background RMS amplitude was thereafter divided by the mean of measured maximal M-wave RMS amplitudes and multiplied by 100.

Peak to peak amplitude and 30 ms RMS were automatically analysed from the MEPs only for biceps brachii. The algorithm used to analyse M-wave was used for MEP analysis as well. The measured MEP peak to peak amplitudes were normalized to the M-wave by dividing the MEP peak to peak amplitude by the mean of M_MAX_ peak to peak amplitudes and multiplying by 100. Similarly, the measured MEP RMS amplitudes were divided by the mean of the measured maximal M-wave RMS amplitudes and multiplied by 100.

SICI was assessed from the MEPs by dividing the mean of the paired-pulse MEPs of a given torque level and task with the respective mean of the single-pulse MEP and by multiplying the ratio by 100. This provides a ratio, where higher values indicate lower SICI.

### Torque steadiness

Standard deviation (SD), coefficient of variation (CV), and median frequency (MDF) of the torque trace of the 500 ms immediately preceding the TMS trigger were analysed as indicators of torque steadiness. SD was defined as the SD of the selected torque epoch. CV was defined as the SD of the selected torque epoch divided by the mean of the selected torque epoch. MDF was assessed with discrete fourier transform (DFT) of the selected torque epoch. The mean of the selected torque epoch was subtracted from the torque and Hann-window was applied to the selected 500 ms epoch prior to applying the DFT. The resulting amplitudes were squared to produce power. MDF was defined as the frequency at which the integral of the power-frequency spectrum from 0 up to that frequeny was equal to or greater than half of the total integral of the power-frequency spectrum.

### Statistical analyses

Unless otherwise noted, all results are reported as means (±SD). Repeated measures multivariate analysis of variance (repeated measures MANOVA) was used to assess the difference between the tasks (i.e., low visuomotor demand task vs. high visuomotor demand task) and between torque levels (i.e., 5, 20, and 40% of MVC torque). Repeated measures ANOVA was used to assess univariate pairwise comparisons between torque levels within a task. Percent differences between force levels and/or tasks are reported by using the value measured at 5% MVC torque as the denominator. Statistical analyses were conducted with SPSS 18.0.1 (SPSS Inc.) software and the significance level was set at *P* ≤ 0.05.

## Results

Mean isometric elbow flexion MVC torque was 27(±10) Nm. Maximal peak to peak M-wave amplitudes were 16.8(±5.9) mV and 5.9(±7.7) mV in biceps brachii and brachioradialis, respectively. The respective values for RMS amplitude were 6.3(±2.4) and 1.7(±2.3) mV.

Multivariate comparison with background sEMG indicated that the tasks did not differ from each other (*F* = 0.4, *P* = 0.68), torque levels did differ (*F* = 8.3, *P* < 0.001) and that there was no task × torque level interaction (*F* = 1.2, *P* = 0.32). Univariate comparisons within task were in line apart from brachioradialis background sEMG RMS amplitude, which did not differ significantly between torque levels (*F* = 2.5, *P* = 0.15). Biceps brachii background sEMG RMS amplitude pairwise comparisons indicated that all of the torque levels significantly differed from each other, with increasing background EMG with increasing torque level (*P* = 0.001; Table 1).

**Table 1:** **Background sEMG from different submaximal torque levels in the high and low visuomotor feedback tasks from the right arm biceps brachii and brachioradialis muscles**.

	**High visuomotor feedback**			**Low visuomotor feedback**		
	**5% MVC**	**20% MVC**	**40% MVC**	**5% MVC**	**20% MVC**	**40% MVC**
Biceps brachii [% M_MAX_]	1.9 (±0.6)	4.9 (±2.6)^a^	10.2 (±5.6)^a^,^b^	2.0 (±0.9)	4.6 (±2.6)^a^	10.4 (±6.4)^a^,^b^
Brachioradialis [% M_MAX_]	9.2 (±12.1)	26.2 (±41.8)	60.3 (±111.3)	9.3 (±11.7)	22.8 (±39.8)	60.5 (±113.9)

Further details of statistical comparisons given in text.

Repeated measures multivariate analysis of variance indicated that high and low visuomotor feedback tasks did not differ from each other and there was no task x torque level interaction. Torque levels did differ from each other.

a p ≤ 0.05 compared to 5% MVC;

b *p ≤ 0.05 compared to 20% MVC*.

Torque steadiness did not differ between tasks (*F* = 2.2, *P* = 0.17), did differ between torque levels (*F* = 33.3, *P* < 0.001) and no task × torque level interaction was observed (*F* = 1.9, *P* = 0.11). Univariate comparisons gave similar results apart from torque SD, which indicated a significant 14% difference between tasks (*F* = 5.7, *P* = 0.04). Pairwise comparisons indicated that all of the torque levels within each torque steadiness variable differed from each other significantly. SD increased with increasing torque levels, CV was the highest at 5% MVC and higher at 40% MVC than at 20% MVC, whereas MDF decreased with increasing torque levels (*P* < 0.001 to *P* = 0.04; Table 2).

**Table 2:** **Torque steadiness from different submaximal torque levels in the high and low visuomotor feedback tasks**.

	**High visuomotor feedback**			**Low visuomotor feedback**		
	**5% MVC**	**20% MVC**	**40% MVC**	**5% MVC**	**20% MVC**	**40% MVC**
Standard deviation [Nm]	0.058 (0.045)	0.086 (0.027)^a^	0.209 (0.071)^a^,^b^	0.056 (0.037)	0.072 (0.038)^a^	0.180 (0.066)^a^,^b^
Coefficient of variation [%]	4.3 (2.5)	1.8 (0.8)^a^	2.1 (0.5)^a^,^b^	4.1 (2.1)	1.5 (0.7)^a^	1.9 (0.8)^a^,^b^
Median frequency [Hz]	7.90 (4.45)	5.03 (1.85)^a^	3.48 (1.51)^a^,^b^	7.37 (3.37)	6.12 (2.82)^a^	3.82 (1.63)^a^,^b^

Further details of statistical comparisons given in text.

Repeated measures multivariate analysis of variance indicated that high and low visuomotor feedback tasks did not differ from each other and there was no task x torque level interaction. Torque levels did differ from each other.

a p ≤ 0.05 compared to 5% MVC;

b p ≤ 0.05 compared to 20% MVC.

**Figure 1:**
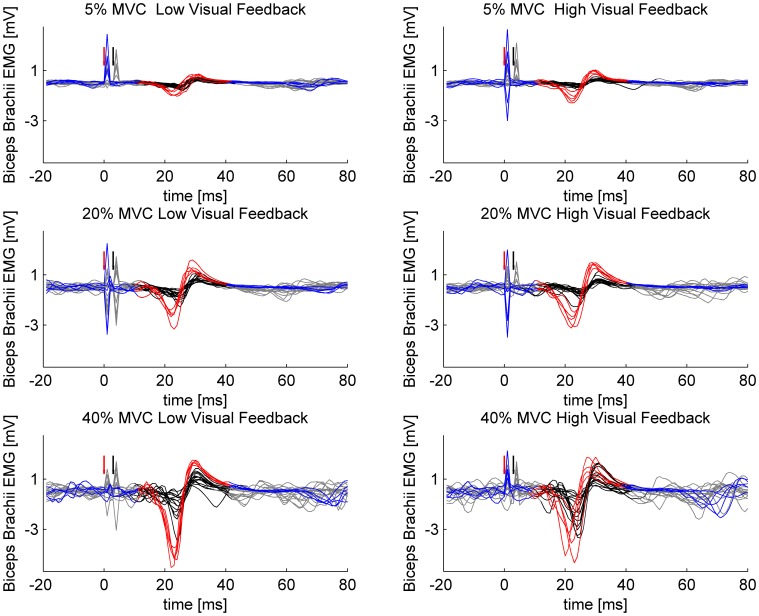
**Sample MEPs from a single participant.** Single- and paired-pulse stimulations are overlaid in different force levels and in different visuomotor feedback tasks. Single-pulse stimuli are plotted with blue and the epoch selected for analysis is highlighted with red. Paired-pulse stimuli are plotted with gray and the epoch selected for analysis is highlighted with black. Red arrow indicates the triggering of single-pulse stimulus or in case of paired-pulse stimulation, the triggering of the conditioning subthreshold (0.8 × AMT) stimulus. Black arrow indicates the triggering of the second suprathreshold (1.2 × AMT) stimulus in paired-pulse stimulations.

**Figure 2:**
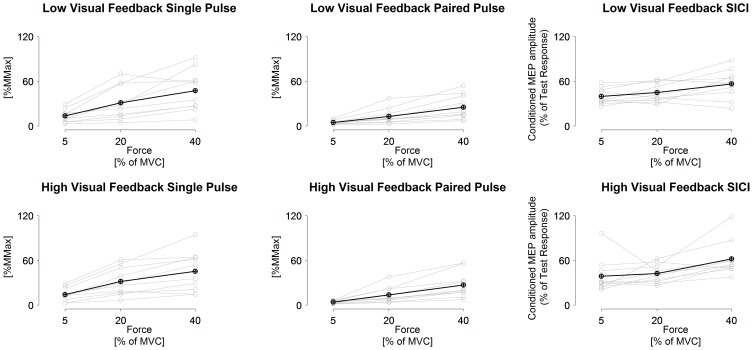
**Measured single-pulse (1.2 × AMT) and paired-pulse (0.8 and 1.2 × AMT with 3 ms inter stimulus interval) MEPs and short-interval intracortical inhibition in low visuomotor and high visuomotor feedback tasks.** Black line, grand mean. Gray lines, results from individual participants. Statistical comparisons given in text.

Multivarate comparisons indicated that single-pulse MEPs did not differ between tasks (*F* = 0.2, *P* = 0.79), did differ between torque levels (*F* = 4.9, *P* = 0.003) and no task × torque level interaction was observed (*F* = 0.8, *P* = 0.54; Table 3). Paired-pulse MEPs differed between tasks (*F* = 7.5, *P* = 0.02) and between torque levels (*F* = 6.9, *P* < 0.001), while no task × torque level interaction was observed (*F* = 2.6, *P* = 0.05) (Figure 1). Univariate comparisons were in line with multivariate comparisons for both single-pulse and paired-pulse MEPs other than paired-pulse MEPs not indicating differences between tasks in either peak to peak (*F* = 0.9, *P* = 0.38) or RMS amplitude (*F* = 3.0, *P* = 0.12). In pairwise comparisons, all torque levels differed from each other in both single-pulse and paired-pulse MEP variables, with increasing MEPs with increasing torque levels (*P* < 0.001 to *P* = 0.01; Table 3 and Figure 2).

**Table 3:** **Single-pulse and paired-pulse motor-evoked potentials (MEP) and short-interval intracortical inhibition (SICI) from different submaximal torque levels in the high and low visuomotor feedback tasks from the right arm biceps brachii muscle**.

	**High visuomotor feedback**			**Low visuomotor feedback**		
	**5% MVC**	**20% MVC**	**40% MVC**	**5% MVC**	**20% MVC**	**40% MVC**
**PEAK TO PEAK**						
Paired-pulse MEP [% M_MAX_]	5.6 (±2.6)	18.1 (±14.2)^a^	35.0 (±21.8)^a^,^b^	6.2 (±3.1)	17.4 (±13.8)^a^	33.6 (±21.2)^a^,^b^
Single-pulse MEP [% M_MAX_]	17.8 (±10.7)	40.4 (±23.5)^a^	57.8 (±30.7)^a^,^b^	16.6 (±9.4)	39.7 (±28.2)^a^	59.6 (±31.9)^a^,^b^
Inhibition [Paired-pulse % of single-pulse]	38.7 (±21.3)	43.8 (±12.5)	62.7 (±24.7)^a^,^b^	39.6 (±8.2)	46.7 (±13.0)^a^	59.7 (±21.4)^a^,^b^
**RMS**						
Paired-pulse MEP [% M_MAX_]	4.3 (±1.9)	13.8 (±10.7)^a^	27.0 (±17.3)^a^,^b^	4.8 (±2.5)	13.1 (±10.4)^a^	25.3 (±16.4)^a^,^b^
Single-pulse MEP [% M_MAX_]	14.3 (±9.0)	31.9 (±18.7)^a^	45.5 (±26.5)^a^,^b^	13.6 (±8.5)	31.3 (±22.8)^a^	47.6 (±27.4)^a^,^b^
Inhibition [Paired-pulse % of single-pulse]	38.8 (±22.7)	42.5 (±12.2)	62.1 (±23.7)^a^,^b^	39.8 (±10.9)	45.1 (±12.7)	56.6 (±19.5)^a^,^b^

Further details of statistical comparisons given in text.

Repeated measures multivariate analysis of variance (MANOVA) indicated that there was no task x torque level interaction. Torque levels differed from each other. For RMS the repeated measures MANOVA indicated a difference between tasks, whereas not for peatk to peak.

a p ≤ 0.05 compared to 5% MVC;

b p ≤ 0.05 compared to 20% MVC.

SICI did not differ between tasks (*F* = 0.9, *P* = 0.43), did differ between torque levels (*F* = 4.7, *P* = 0.004) and no task × torque level interaction was observed (*F* = 2.1, *P* > 0.10). Univariate comparisons were in line with the multivariate comparison. Pairwise comparisons indicated that the highest torque level had less inhibition than the two lower torque levels in both RMS and peak to peak amplitude derived SICI (*P* = 0.01 to 0.05), while 5% MVC had less inhibition than 20% MVC only in the low visual feedback task in peak to peak amplitude derived SICI (*P* = 0.04; Table 3 and Figure 2).

## Discussion

The primary findings of the present study were that SICI decreased with increasing torque levels, but was unaffected by the change in the visual feedback. Furthermore, there were no interaction effects between task and torque level. The finding that SICI decreases with increasing force supports our hypothesis and is consistent with what has previously been observed in hand muscles (Zoghi and Nordstrom, 2007; Ortu et al., 2008). However, the finding that the change in the visual feedback did not affect SICI is in contrast to our hypothesis.

The finding that SICI decreases with increasing force is consistent with the findings of Zoghi and Nordstrom (2007), who showed that SICI decreases from 5 to 25% MVC in the first dorsal interosseus (FDI) (Zoghi and Nordstrom, 2007). This was also supported by Ortu et al. (2008), who found that SICI was decreased when the force level was increased from 10 to 25% and 50% MVC in the FDI (Ortu et al., 2008). Single-pulse MEPs are known to increase up to around 50% of MVC in biceps brachii (Kamen, 2004; Martin et al., 2006), as was also the case in the present study. Force gradation is executed by recruiting new motor units and by increasing the firing frequency of the already active motor units (Masakado, 1994). Taken together, the findings indicate that SICI is modulated to a meaningful magnitude in torque levels at least up to 40% MVC (nota bene higher torque levels were not measured in the present study), and contributes to changes in corticospinal excitability in biceps brachii. Interestingly, whilst no additional disinhibition was reported in FDI by Ortu et al. (2008) from 10% MVC or above, the present study demonstrated the least inhibition at the highest torque level (40% MVC). This difference could be attributed to differences in muscles tested, as FDI has fewer motor units than biceps brachii (Enoka, 1995). Consequently, new motor units are recruited up to 90% of MVC in biceps brachii (Kukulka and Clamann, 1981), but only 50% of MVC in FDI (Luca et al., 1982). This characteristic is considered typical for smaller muscles with fewer motor units (Masakado, 1994). Thus, we speculate that the role of SICI in force gradation may be more pronounced in the determination of whether new motor units are recruited than in rate coding. The speculative mechanism would be by modifying the excitability of pyramidal cells.

The paradigm used to modify the visual feedback scaling in the present study was very similar to one we have applied previously for hand muscles, in which changes in silent period were observed (Pearce and Kidgell, 2009, 2010). Silent period and SICI are mediated by two different mechanisms (Chen et al., 2008) and consequently it is plausible that the changes observed in silent period in previous studies (Hess et al., 1999; Pearce and Kidgell, 2009, 2010) have not been SICI related either. In line with the present study, comparing different kinds of grips (power grip, precision grip) or different tasks (isometric abduction of index finger vs. precision grip) have not indicated SICI to be preferentially modulated when the background sEMG levels of the muscle of interest have been held constant (Christova et al., 2006; Davare et al., 2008; Kouchtir-Devanne et al., 2012). However, changes in corticospinal excitability, as assessed with MEP amplitude or input/output curves, have been reported (Christova et al., 2006; Kouchtir-Devanne et al., 2012). M1 receives cortical connections from other cortical areas (Davare et al., 2011), such as somatosensory cortex and pre-motor area (Ghosh and Porter, 1988), which are not necessarily reflected in SICI. It is known that intracortical facilitation and inhibition are mediated with different groups of neurons and can be modulated independently (Chen et al., 2008). Keeping the above in mind, it is speculated that SICI has a more prominent role in force gradation than in task-dependent coordination, which may be modulated by other neural circuits at the cortical level.

It should be noted that previous studies using visuomotor feedback to modify force steadiness have demonstrated significant changes in force steadiness when force is produced with or without visuomotor feedback (Tracy, 2007; Tracy et al., 2007). Moreover, changes in force steadiness have been reported when visuomotor feedback is varied over a wide range of visual scales (Sosnoff and Newell, 2006). While multivariate analysis did not show differences in torque steadiness in the present study, torque SD did indicate a difference between the tasks in univariate analysis.

There are some limitations in the present study that need to be highlighted. Only a single block of five single-pulse TMS stimuli were recorded in each task at each force level to keep the duration of the experiment within a feasible time frame. However, the randomization of single-pulse and paired-pulse stimuli was likely to sufficiently minimize any systematic effects introduced by the order of the trials. Input/output curves have proven sensitive in identifying changes in corticospinal excitability in comparisons with different tasks, however, input/output curves were not measured in the present study. For example, Kouchtir-Devanne et al. (2012) compared index finger abduction vs. precision grip and found that SICI did not differ between tasks, wheareas corticospinal excitability as assessed with input/output curves did (Kouchtir-Devanne et al., 2012). We did not, however, observe differences in MEPs between tasks, which appears to indicate that corticospinal excitability did not differ between tasks in the present study.

In conclusion, SICI was not modulated in a task-dependent manner in tasks with differing visual feedback. SICI was, however, decreased with increasing submaximal torque providing further evidence that modifying SICI is an important mechanism for modulating cortical excitability and plays a role in force gradation.

### Conflict of interest statement

The authors declare that the research was conducted in the absence of any commercial or financial relationships that could be construed as a potential conflict of interest.
